# Interrelationships of Parenting Information, Family Care, and Child Development: A Cross-Sectional Study in Rural China

**DOI:** 10.3390/ijerph17165737

**Published:** 2020-08-08

**Authors:** Jingdong Zhong, Renfu Luo

**Affiliations:** 1 School of Economics, Peking University, Beijing 100871, China; jdzhong@pku.edu.cn; 2 China Center for Agricultural Policy, School of Advanced Agricultural Sciences, Peking University, Beijing 100871, China

**Keywords:** early childhood development, parenting information, family care, rural China

## Abstract

This paper studied the interrelationships between parenting information, family care, and early childhood development (ECD) outcomes. A total of 1787 sample households in rural China were enrolled in a cross-sectional study. A demographic questionnaire, a parenting information questionnaire, the Family Care Indicators (FCIs), and the Bayley Scales of Infant Development version III (BSID-III) were used to measure demographic characteristics, parenting information that the caregiver received, family care, and early development outcomes of the child, respectively. Structural equation modeling (SEM) was then used to estimate the interrelationships. The results showed that family care significantly mediated between parenting information and ECD outcomes. Through family care, one standard deviation (SD) increase in the parenting information was associated with the increase in the child’s four development outcomes (cognition, language, motor, and social–emotion) by 3%, 4%, 4%, and 5% of one SD, respectively. Different measurements of parenting information and different components of family care played different roles in the interrelationships. The key findings of this study are informative for providing early child development services in rural China.

## 1. Introduction

Drastic delays in early childhood development (ECD) is a noteworthy problem among children across rural areas in China. As estimated by Wang et al. [1], 85% of the children who are 0–3 years old in four major subpopulations of rural China do not reach their full potential in at least one kind of development outcome. Especially, 49%, 52%, 30%, and 53% of the children were delayed in cognitive development, language development, motor development, and social–emotional development, respectively.

Undoubtedly, such early delays are detrimental to these children’s lifetime outcomes, as a growing body of literature has documented the importance of ECD outcomes in many aspects, such as health [2,3], labor market performance [4,5], social mobility [6], and other socioeconomic status (SES) in adulthood [7,8]. The development delay in early childhood was identified as a potential identifier of the middle-income trap, which could affect sustainable economic development [9].

Early development delays are usually accompanied by poor family care in rural households in China. Family care encompasses the provision of a safe, clean, and stimulating home environment that supports early childhood development [10]. For one thing, rural caregivers provide very few play materials for the children in the family [11,12]. Rural caregivers also seldom engage in interactive parenting activities [13,14,15]. In rural China, only 12.6% of caregivers read with their children [13] and only 13.8% tell stories to their children [14]. On average, rural children play alone for about 2.5 h per day, implying the absence of caregiver-child interaction in the family [15]. For another thing, family care is significantly and positively associated with ECD outcomes [14,15,16,17,18]. In rural China, the children’s cognitive and language development measured by the Mental Development Index in the Bayley Scales of Infant Development version I (BSID-I) was significantly higher by 0.48 standard deviation (SD), 0.51 SD, and 0.34 SD on average when their caregivers told stories to them, sang songs to them, and used the play materials to play with them, respectively; the children’s psychomotor development measured by the Psychomotor Development Index in the BSID-I was also significantly higher by 0.20 SD, 0.27 SD, and 0.18 SD on average when their caregivers told stories to them, sang songs to them, and used the play materials to play with them, respectively [15]. Similar results were found in other areas in rural China too [14]. Previous research from other countries also found strong links between positive parenting (reading, storytelling, and playing) and improved ECD outcomes [16,17,18].

Poor family care could partly originate from the lack of parenting information. Parenting information refers to the information about positive parenting practices, that the caregivers could engage in, to help the child to reach their full development potential [19]. In the USA, mothers with low SES have fewer sources of parenting information, and their frequency of sharing reading with their children in infancy is lower than those with high SES [19]. Similarly, in rural China, many caregivers never receive the information to help them to create a stimulating home environment, so they do not know how to successfully stimulate their children’s development [15,20]. Furthermore, some parenting programs that delivered parenting information to caregivers in rural areas found that, in the intervention group, family care in terms of parental investments increased by 0.35–0.72 standard deviation (SD), and the child’s cognitive development increased by 0.23–0.27 SD [21,22]. These findings indicated that family care might mediate in the links between parenting information and child development. To date, however, the interrelationships have not yet been estimated by a structural model.

The overall objective of this study was to investigate the interrelationships of parenting information, family care, and early child development in rural China. This study had the following three specific objectives: first, identifying whether family care mediates between parenting information and ECD outcomes; second, estimating the indirect effects of different measurements of parenting information through family care; and third, estimating the mediation effects of different components of family care.

To achieve these objectives, this study proposed the corresponding study hypotheses as follows: first, family care plays the mediator role between parenting information and ECD outcomes; second, different measurements of parenting information has different indirect effects through family care; and third, the mediation effects of different components of family care vary across development outcomes.

This study contributes to the existing work [13,14,15,16,17,18] by identifying the mediation effects of family care on the relationships between parenting information and early child development, and the heterogeneity across different measurements of parenting information and different components of family care. This study provides information for public policy makers and provides data that can serve as a foundation for providing early child development services in rural China.

## 2. Methods

### 2.1. Participants

The study was conducted in 22 poverty-stricken rural counties in a relatively undeveloped province located in northwestern China. The per capita income of this province ranked below the median among all provinces of China in 2016.

The following three-step protocol was used to choose the study participants. First, a list of all 245 towns in the sample counties was obtained from the local population and health authorities. A random computerized number generator was used to randomly choose 118 towns from the list, based on the sample size calculated for a randomized controlled trial. Second, one village was randomly selected into the baseline survey within each sample town. Third, within each sample village, based on the list of all registered births from the local official, all households with children aged 6–24 months old were sampled in the study.

All 1788 households invited to participate in the study agreed to do so. One sample household, however, did not finish the interview; thus, there were 1787 sample households in total in the final analysis.

Before participating in the study, all participants gave their informed consent for inclusion. The study was in accordance with the Declaration of Helsinki. The Ethics Committee of Stanford University, Stanford, CA, USA, approved the study (No. 35921).

### 2.2. Data Collection

In the 2016 field survey, four types of information were collected from the sample households: (1) demographic characteristics, (2) parenting information, (3) family care, and (4) early child development. During the fieldwork, the trained enumerators made a 90–120 min long home visit when the caregiver was present and the child was awake at home. All measurements were performed at the initial home visit that occurred right after the sampling, i.e., all the sample children were 6–24 months old then.

The following survey instruments were used to collect the data:

(1) Demographic questionnaire. The primary caregiver of each sample child was identified as the one who takes the most responsibility on the child’s daily care. The demographic questionnaire was completed by the primary caregiver, which consisted of the child’s gender, the child’s age, whether the child was born with low weight (child’s birthweight lower than 2500 g), the caregiver’s age, the caregiver’s education, and whether the mother was the child’s primary caregiver.

(2) Parenting information questionnaire. The questionnaire measuring parenting information was administered to each primary caregiver. As shown in Table A1, there are eight items in the questionnaire: “the caregiver was told about how to teach the child to keep away from danger (such as pesticides, pond, and fire)”; “the caregiver was told about how to teach the child to self-care (such as brushing teeth and wearing clothes)”; “the caregiver was told about how to teach the child to understand and to use words”; “the caregiver was told about how to read books with the child”; “the caregiver was told about how to sing songs with the child”; “the caregiver was told about how to play games with the child”; “the caregiver was told about how to teach the child to get along with peers”; and “the caregiver was told about how to teach the child to understand and to obey the rules”. Caregivers responded to these items by the 0–1 binary choice (1 = yes; 0 = no). The Cronbach’s alpha coefficient of the questionnaire is 0.88, indicating that it has adequate internal consistency in the sample [23].

(3) Family Care Indicators (FCIs). The FCIs, developed by the UNICEF experts to measure family care [24], yield both validity and reliability [25]. The inventory has been formally translated into the Chinese language to adapt to the context in rural areas [11,12]. It was administered to each primary caregiver. As shown in Table A2, the inventory contains a total of 19 items in five subscales. In three subscales (“sources of play materials”, “varieties of play materials”, and “play activities”), the items were scored by the 0–1 binary choice (1 = yes; 0 = no). In the remaining two subscales (“household books” and “magazines and newspapers”), the items were scored by the four-point scale based on the quantity (1 = “none”; 2 = “1–2”; 3 = “3–5”; 4 = “≥6”). The Cronbach’s alpha coefficient of the inventory is 0.75, indicating that it has adequate internal consistency in the sample [23]. The subscale scores were calculated by summing up the scores of relevant items.

(4) Bayley Scales of Infant Development version III (BSID-III). The BSID-III, designed by Bayley [26] to access the development of children under age three, is an internationally-used golden-standard instrument. It has been formally translated into the Chinese language to adapt to the context in rural areas [1]. In the cognitive, language, and motor subscale, the scores are based on the child’s successful completion of the tasks. In the social-emotional subscale, the score is based on the caregiver’s responses to questions developed from the Greenspan Social-Emotional Growth Chart [27]. All enumerators had taken a week-long training course on how to administer the test before the fieldwork, but they were blind to the study. In the fieldwork, the trained enumerators used a detailed scoring sheet and a standardized set of toys to administer the test for each sample child when the caregiver was present, but the caregiver was not allowed to help the child. The subscale reliability coefficients are all above 0.8, indicating that they have adequate internal consistency in the sample [23].

### 2.3. Statistical Analysis

**Figure 1:**
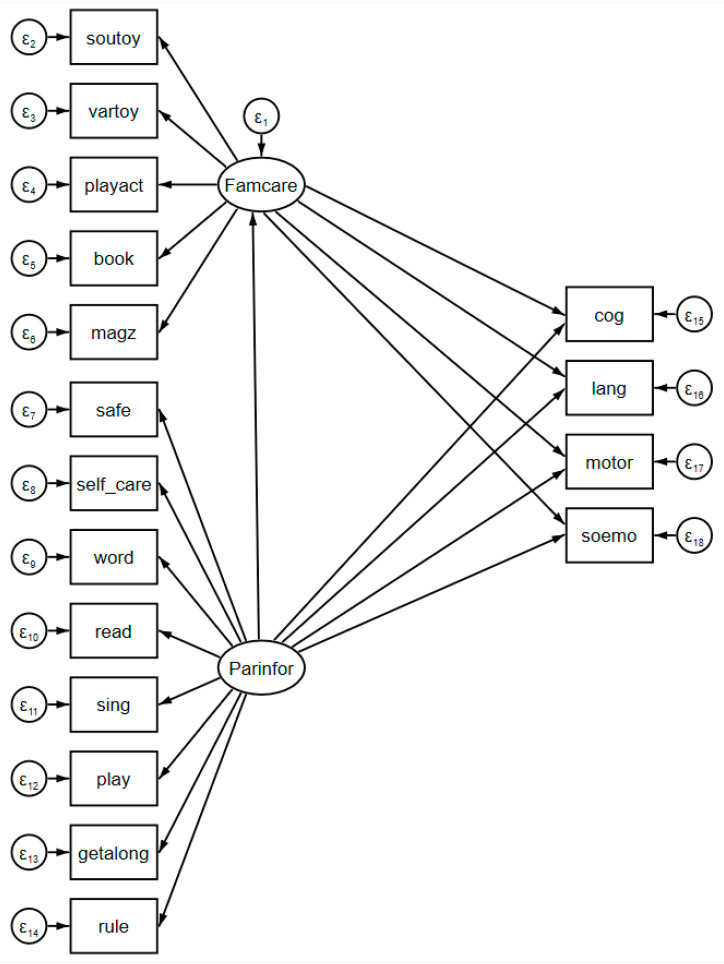
Structural equation modeling (SEM) for the mediation model.

Following Preacher et al. [28], structural equation modeling (SEM) was used to depict the interrelationships between parenting information, family care, and child development, as shown in Figure 1.

Figure 1 was drawn by using the SEM builder in the Stata 15.0 software (StataCorp LLC, College Station, TX, USA). The independent variable was the latent variable (Parinfor) of observed parenting information measures (safe, self_care, word, read, sing, play, getalong, and rule). The dependent variables were the child’s BSID-III scores in four development outcomes (cog, lang, motor, and soemo). The mediator was the latent variable (Famcare) of observed family care measures (soutoy, vartoy, playact, book, and magz). The definitions of these variables are shown in Table 1.

In the measurement component, all observed parenting information measures were used to construct a latent variable of parenting information (Parinfor), and all observed family care measures were used to construct a latent variable of family care (Famcare). In the mediation component, the dependent variable was the child’s four subscale scores in the BSID-III test; the independent variable was the latent variable of parenting information (Parinfor); and the mediator was the latent variable of family care (Famcare). The maximum likelihood (ML) method was used to estimate the model.

Then, the estimates were adjusted by the control variables, which included the demographic characteristics (the child’s gender, the child’s age, whether the child was born with low weight, the caregiver’s age, the caregiver’s education, and whether the mother was the child’s primary caregiver), and the county fixed effects (county FE) that account for the unobserved county heterogeneity.

Furthermore, to examine what kind of parenting information helps the caregiver more, the eight observed parenting information measures were used to replace the latent variable (Parinfor) as the independent variables in the model, and indirect effects of different measurements of parenting information through family care were estimated based on the SEM with control variables. Similarly, to examine which dimension of family care strongly mediates, the five observed family care measures were then used to replace the latent variable (Famcare) as mediators, and the indirect effects through these mediators were estimated again.

Following Preacher and Hayes [29], the bootstrap method based on resampling with 1000 replications was used to calculate the standard errors (S.E.) of the indirect effects. To test the statistical significance of the indirect effects, three types of 95% confidence interval (CI), including the percentile CI, the bias-corrected (BC) CI, and the bias-corrected and accelerated (BCa) CI, were calculated. The indirect effect was statistically significant if the CIs did not contain the zero. The statistical software Stata 15.0 was used for the statistical analysis.

## 3. Results

### 3.1. Descriptive Statistics

Table 1 reports the summary statistics of all observed measures in the sample. In terms of child development, the mean ± SD of four development scores for cognition, language, motor, and social–emotion in the BSID-III test were 95.97 ± 12.55, 92.47 ± 13.50, 97.29 ± 16.48, and 86.04 ± 15.29, respectively (rows 1–4). In terms of family care (rows 5–9), the mean ± SD of sources of play materials, varieties of play materials, play activities, books, and magazines and newspapers in the households were 2.36 ± 1.01, 3.76 ± 1.86, 2.68 ± 1.65, 2.37 ± 1.29, and 1.61 ± 1.02, respectively.

In terms of parenting information (rows 10–17), caregivers were told about the relevant parenting information on how to teach the child to keep away from danger (49%), how to teach the child to self-care (37%), how to teach the child to understand and to use words (35%), how to read a book with the child (30%), how to sing songs with the child (36%), how to play games with the child (43%), how to teach the child to get along with peers (37%), and how to teach the child to understand and to obey the rules (39%). In terms of demographic characteristics, on average, 52% of the children were male (row 18), children were less than 15 months old (row 19), 4% of the children were born with low weight (row 20), caregivers were slightly over 35 years old (row 21), caregivers completed about eight years of education (row 22), and finally, the child’s mother was the primary caregiver in 69% of the sample households (row 23).

### 3.2. Interrelationships between Parenting Information, Family Care, and Child Development

Table 2 reports the estimates of the mediation model. Panel A shows the unadjusted estimates based on the SEM without control variables. The direct effects of parenting information on the child’s four development outcomes (cognition, language, motor, and social-emotion) were not statistically significant at the 5% significance level (row 1, columns 1–4). However, family care was positively associated with all four outcomes at the 1% level (row 2, columns 1–4). Parenting information was also positively associated with family care at the 1% level (row 1, column 5).

Panel B shows the adjusted estimates based on the SEM with control variables, and the results were identical to the unadjusted ones. The direct effects of parenting information on child development were not statistically significant at the 5% level (row 3, columns 1–4). A one SD increase in the family care was significantly associated with the increase in the four development outcomes by 0.12 SD, 0.12 SD, 0.12 SD, and 0.17 SD at the 1% level, respectively (row 4, columns 1–4). In the meantime, the increase in the parenting information by one SD significantly corresponded to the increase in family care by 0.30 SD at the 1% level (row 3, column 5).

### 3.3. Mediation Effects of Family Care

Table 3 reports the estimated indirect effects of the parenting information on child development through family care. Panel A shows the unadjusted estimates. Point estimates of indirect effects on four development outcomes were all significantly larger than zero at the 1% level (rows 1–4, column 1). The corresponding 95% CIs did not contain zero (rows 1–4, columns 3–5), which further suggested that family care indeed has significant mediation effects on the relationships between parenting information and ECD outcomes.

Panel B shows the adjusted estimates that were identical to the unadjusted estimates. Through family care, one standard deviation (SD) increase in the parenting information was significantly associated with the increase in the child’s four development outcomes (cognition, language, motor, and social-emotion) by 3%, 4%, 4%, and 5% of one SD, respectively (rows 5–8).

Table 4 reports the estimated indirect effects of different measurements of parenting information on child development through family care. For the child’s cognitive development (Panel A), the indirect effects of parenting information on how to read a book with the child (row 4), how to teach the child to get along with peers (row 7), and how to teach the child to understand and to obey the rules (row 8) were the largest, with the effect sizes of 0.02 SD, followed by parenting information on how to teach the child to self-care (row 2), how to sing songs with the child (row 5), and how to play games with the child (row 6), with the effect sizes of 0.01 SD. The indirect effects of the other two measurements of parenting information, however, were not statistically significant (rows 1 and 3). For the child’s language development (Panel B), motor development (Panel C), and social-emotional development (Panel D), the results were identical: the six measurements of parenting information had significant indirect effects through family care.

Table 5 reports the estimated indirect effects of parenting information on child development through different components of family care. For child’s development in cognition (Panel A), language (Panel B), and motor (Panel C), the variety of play materials was the only significant mediator, through which a one SD increase in the parenting information was associated with a 0.02 SD increase in the child’s cognitive, language, and motor score at the 1% level (rows 2, 7, and 12). For child’s development in social-emotion (Panel D), the number of play activities was the strongest mediator (row 18), followed by the variety of play materials (row 17) and the number of household books (row 19), through which a one SD increase in the parenting information was significantly associated with the increase in the child’s social-emotional score by 0.03 SD, 0.02 SD, and 0.01 SD, respectively.

## 4. Discussion

This paper investigated the interrelationships between parenting information, family care, and ECD outcomes in rural households. Family care strongly mediated between parenting information and the child’s four development outcomes. Six measurements of parenting information were significantly associated with child development through family care. The mediation effects of different components of family care also varied across development outcomes. The variety of play materials had significant mediation effects for all four development outcomes of the child, while the number of play activities and the number of household books only significantly mediated for the child’s social-emotional development.

As found by a growing body of literature, a child’s early development could bring high returns to his/her welfare in adulthood [2,3,4,5,6,7,8] and the long-term economic growth of the country [9]. The key findings of this paper strongly indicated that delivering the information on positive parenting practices to the caregiver is indeed beneficial to the child’s ECD outcomes, which is in line with the findings of Sylvia et al. [21] and Luo et al. [22].

A large share of rural caregivers did not know how to successfully create a stimulating home environment for child development [20]. The findings of this paper further suggested that a lack of relevant parenting information is one source of the poor home environment in rural households since family care plays a key mediator role between parenting information and early child development. More parenting information on the caregiver-child interactions, such as reading books, singing songs, and playing games with the child, and other positive practices, such as teaching the child to get along with peers, and to understand and obey the rules, would help the caregiver to improve the quality of family care for the child. This is in line with the existing pieces of evidence that more parenting information would be accompanied by more positive parenting practices of the caregiver related to early development stimulation, such as telling stories to the child, singing to the child, and using play materials to play with the child, in both developed countries [16,17,19] and developing countries [15,21,22]. In the meantime, the improved family care would lead to better ECD outcomes of the child. This is also consistent with the existing evidence on the positive associations between family care and child development including cognitive and non-cognitive outcomes, such as personality and behaviors [30,31].

The findings also revealed that different components of family care play heterogeneous roles between parenting information and development outcomes. On the one hand, play materials are important material inputs for child development [11,12,32,33]. The caregiver who has received more parenting information would prepare more varieties of play materials in the household, which in turn, foster the child’s development in all four ECD outcomes. However, the mediation effects of sources of play materials were not statistically significant. This is in line with Hamadani et al. [25], which found that, in Bangladesh, sources of play materials were not significantly associated with the child’s early development outcomes.

On the other hand, interactive play activities are productive time inputs for child development, in both developed contexts [16,17,34,35] and developing contexts [13,14,15]. The caregiver who has received more parenting information would engage in more play activities, which correspond to the significant improvement in the child’s social-emotional development. A child’s social-emotional development during early childhood could foster his/her cognitive development in adolescence [36], and thus has more lasting effects on his/her adulthood welfares [31]. This adds to the existing evidence on the importance of the learning-by-playing at the early stage for child development [37].

According to Yue et al. [15], rural caregivers who received the parenting information from professional sources, such as local doctors and local health organization, were more likely to tell stories to their children, sing songs to their children, and play with their children using play materials than those who received the parenting information from nonprofessional sources, such as friends, TV, books, and the Internet. Sylvia et al. [21] and Luo et al. [22] showed that a home-based parenting curriculum delivered by workers from the public service system could effectively promote caregiver’s parenting behaviors and child’s cognitive development in rural China. Previous research in rural China suggested that the professional parenting curriculum could be useful as educational material to deliver the parenting information to the caregivers, although this study lacked identification regarding this in detail.

Further to this, as for the caregiver’s literacy level measured by the completed year of schooling, this study found that the caregiver’s more years of schooling were significantly and positively associated with the quality of family care and the child’s early development. This indicated that, with higher literacy levels, the caregivers would have a stronger ability to be able to comply with delivering an educational program (or its contents) to their children. This is consistent with the existing pieces of evidence that more educated parents would more frequently engage with the child in parenting activities, and thus benefit a child’s early development [31,38].

## 5. Strengths and Limitations

A strength of this study was the enrollment of children and caregivers from rural households in western China. The demographic characteristics of the study sample in the paper were comparable to those of the sample households collected from other rural areas of western China by other studies [1].

However, we acknowledge that this study faced several limitations. First, the sample data was collected from only one rural area in western China, so the study sample was not representative of the general population across rural areas, and the findings may not be generalizable to other contexts. Second, as the sample children were somehow young (less than 15 months old) at the field survey, a few measures in the parenting information questionnaire used in this study should be more age-appropriate for the children at that age. Third, given the nature of the cross-sectional study, the SEM estimation of the mediation model did not necessarily state the causal inference, even though they were useful to understand the interrelationships. Fourth, this study did not identify the importance of nutrition on children in the early age group. Previous research had already found that across rural China, nearly one-third to one-half of children did not have adequate micronutrient intake, which was identified as one contributing factor of early development delays [1,39]. Fifth, although this study discussed some information about the sources of educational materials, it did not indicate consistency in the material offered to caregivers.

This study also offered some broad perspectives on future research. First, future research could collect more representative sample data in terms of the general population in rural China, so as to draw more generalizable conclusions that could be applied across rural areas. Second, future research could design a more age-appropriate, stage-based questionnaire to better access the caregiver’s parenting information. Third, future investigation based on a longitudinal study could be helpful to examine the causal links between parenting information, family care, and early child development. Fourth, further identification regarding what the educational materials supplied to caregivers could be valuable too.

## 6. Conclusions

In conclusion, this paper demonstrated that family care strongly mediates between parenting information and ECD outcomes in rural households. The key findings of this study have important policy implications. Targeted interventions to deliver parenting information to the caregiver at a child’s early age (around 15 months old) are effective to improve family care for the child and benefit the child’s development. Early interventions to increase the varieties of play materials and the play activities in the households could be helpful for early childhood development too.

## Figures and Tables

**Table 1:** Summary statistics (*N* = 1787 for all variables).

Variable	Definition	Mean ± SD
**Child development**		
(1) cog	BSID-III cognitive score	95.97 ± 12.55
(2) lang	BSID-III language score	92.47 ± 13.50
(3) motor	BSID-III motor score	97.29 ± 16.48
(4) soemo	BSID-III social-emotional score	86.04 ± 15.29
**Family care**		
(5) soutoy	sources of play materials	2.36 ± 1.01
(6) vartoy	varieties of play material	3.76 ± 1.86
(7) playact	play activities	2.68 ± 1.65
(8) book	household books	2.37 ± 1.29
(9) magz	magazines and newspapers	1.61 ± 1.02
**Parenting information**		
(10) safe	1 = caregiver was told about how to teach the child to keep away from danger (such as pesticides, pond, and fire), 0 = no	0.49 ± 0.50
(11) self_care	1 = caregiver was told about how to teach the child to self-care (such as brushing teeth and wearing clothes), 0 = no	0.37 ± 0.48
(12) word	1 = caregiver was told about how to teach the child to understand and to use words, 0 = no	0.35 ± 0.48
(13) read	1 = caregiver was told about how to read books with the child, 0 = no	0.30 ± 0.46
(14) sing	1 = caregiver was told about how to sing songs with the child, 0 = no	0.36 ± 0.48
(15) play	1 = caregiver was told about how to play games with the child, 0 = no	0.43 ± 0.50
(16) getalong	1 = caregiver was told about how to teach the child to get along with peers, 0 = no	0.37 ± 0.48
(17) rule	1 = caregiver was told about how to teach the child to understand and to obey the rules, 0 = no	0.39 ± 0.49
**Demographic characteristics**		
(18) male	1 = male, 0 = no	0.52 ± 0.50
(19) month	child’s age in months	14.44 ± 5.40
(20) lbw	1 = child has low birthweight, 0 = no	0.04 ± 0.20
(21) cage	caregiver’s age	35.38 ± 12.28
(22) cedu	caregiver’s completed year of schooling	8.05 ± 3.32
(23) momcare	1 = mother is the primary caregiver, 0 = no	0.69 ± 0.46

**Table 2:** SEM estimation of the mediation model (*N* = 1787).

	Cog	Lang	Motor	Soemo	Famcare
	(1)	(2)	(3)	(4)	(5)
**Panel A. Unadjusted estimates**					
(1) Parinfor	0.03 (0.02)	−0.005 (0.03)	0.01 (0.02)	−0.02 (0.03)	0.36 *** (0.02)
(2) Famcare	0.13 *** (0.02)	0.18 *** (0.02)	0.20 *** (0.02)	0.16 *** (0.03)	
**Panel B. Adjusted estimates**					
(3) Parinfor	0.03 (0.03)	0.01 (0.03)	0.02 (0.02)	−0.01 (0.03)	0.30 *** (0.02)
(4) Famcare	0.12 *** (0.03)	0.12 *** (0.02)	0.12 *** (0.02)	0.17 *** (0.03)	
(5) male	−0.06 (0.04)	−0.23 *** (0.04)	−0.04 (0.04)	−0.005 (0.05)	−0.05 (0.04)
(6) month	−0.005 (0.004)	0.03 *** (0.004)	0.09 *** (0.003)	0.002 (0.005)	0.03 *** (0.003)
(7) lbw	−0.40 *** (0.15)	−0.29 ** (0.14)	−0.24 ** (0.11)	−0.34 ** (0.15)	−0.04 (0.10)
(8) cage	−0.001 (0.004)	−0.0001 (0.004)	−0.004 (0.003)	0.002 (0.003)	0.01 ** (0.004)
(9) cedu	0.02 *** (0.01)	0.03 *** (0.01)	0.02 *** (0.006)	0.001 (0.01)	0.08 *** (0.01)
(10) momcare	−0.22 ** (0.10)	−0.06 (0.10)	−0.26 *** (0.08)	−0.19 ** (0.08)	0.19 ** (0.09)
(11) County FE	Yes	Yes	Yes	Yes	Yes

Notes: The maximum likelihood (ML) method was used to estimate the model. Standardized coefficients are presented in the table, and bootstrap standard errors based on resampling with 1000 replications are reported in parentheses. *** *p* < 0.01; ** *p* < 0.05.

**Table 3:** Estimates of indirect effects of parenting information on child development through family care.

Indirect Effect	Point Estimate	Bootstrap S.E.	95% CI (Percentile)	95% CI (BC)	95% CI (BCa)
	(1)	(2)	(3)	(4)	(5)
**Panel A. Unadjusted estimates**					
(1) Parinfor on cog through Famcare	0.05 ***	0.01	(0.03, 0.07)	(0.03, 0.07)	(0.03, 0.07)
(2) Parinfor on lang through Famcare	0.07 ***	0.01	(0.05, 0.08)	(0.05, 0.08)	(0.05, 0.08)
(3) Parinfor on motor through Famcare	0.07 ***	0.01	(0.05, 0.09)	(0.06, 0.10)	(0.06, 0.10)
(4) Parinfor on soemo through Famcare	0.06 ***	0.01	(0.04, 0.08)	(0.04, 0.08)	(0.04, 0.08)
**Panel B. Adjusted estimates**					
(5) Parinfor on cog through Famcare	0.03 ***	0.01	(0.02, 0.05)	(0.02, 0.04)	(0.02, 0.05)
(6) Parinfor on lang through Famcare	0.04 ***	0.01	(0.02, 0.05)	(0.03, 0.05)	(0.03, 0.06)
(7) Parinfor on motor through Famcare	0.04 ***	0.01	(0.02, 0.05)	(0.03, 0.06)	(0.03, 0.06)
(8) Parinfor on soemo through Famcare	0.05 ***	0.01	(0.04, 0.07)	(0.03, 0.07)	(0.03, 0.07)

Notes: The dependent variables are the child’s Bayley Scales of Infant Development version III (BSID-III) scores in four development outcomes (cog, lang, motor, and soemo). The independent variable was the latent variable (Parinfor) measuring parenting information that the caregiver was told about in the past year. The mediator was the latent variable (Famcare) measuring family care for the child. Bootstrap standard errors (S.E.) reported in column (2) were based on resampling with 1000 replications. Confidence interval, CI; bias-corrected, BC; bias-corrected and accelerated, BCa; *** *p* < 0.01.

**Table 4:** Estimates of indirect effects of different measurements of parenting information on child development through family care.

Indirect Effect	Point Estimate	Bootstrap S.E.	95% CI (Percentile)	95% CI (BC)	95% CI (BCa)
	(1)	(2)	(3)	(4)	(5)
**Panel A. Dependent variable is the child’s cognitive score**					
(1) safe on cog through Famcare	−0.005	0.006	(−0.01, 0.006)	(−0.02, 0.004)	(−0.02, 0.005)
(2) self_care on cog through Famcare	0.01 **	0.007	(0.003, 0.02)	(0.003, 0.04)	(0.003, 0.04)
(3) word on cog through Famcare	0.01	0.006	(−0.003, 0.02)	(−0.002, 0.02)	(−0.002, 0.02)
(4) read on cog through Famcare	0.02 ***	0.007	(0.008, 0.03)	(0.01, 0.03)	(0.01, 0.03)
(5) sing on cog through Famcare	0.01 **	0.006	(0.003, 0.03)	(0.004, 0.03)	(0.004, 0.03)
(6) play on cog through Famcare	0.01 **	0.007	(0.001, 0.02)	(0.003, 0.03)	(0.003, 0.03)
(7) getalong on cog through Famcare	0.02 **	0.007	(0.007, 0.03)	(0.008, 0.03)	(0.008, 0.03)
(8) rule on cog through Famcare	0.02 **	0.007	(0.007, 0.03)	(0.008, 0.04)	(0.008, 0.04)
**Panel B. Dependent variable is the child’s language score**					
(9) safe on lang through Famcare	−0.005	0.007	(−0.02, 0.008)	(−0.02, 0.003)	(−0.02, 0.003)
(10) self_care on lang through Famcare	0.01 **	0.007	(0.003, 0.03)	(0.003, 0.03)	(0.003, 0.03)
(11) word on lang through Famcare	0.01	0.007	(−0.004, 0.02)	(−0.004, 0.03)	(−0.004, 0.03)
(12) read on lang through Famcare	0.02 ***	0.007	(0.007, 0.03)	(0.01, 0.04)	(0.01, 0.04)
(13) sing on lang through Famcare	0.01 **	0.007	(0.004, 0.03)	(0.004, 0.03)	(0.004, 0.03)
(14) play on lang through Famcare	0.01 **	0.008	(0.001, 0.02)	(0.002, 0.03)	(0.002, 0.03)
(15) getalong on lang through Famcare	0.02 **	0.007	(0.008, 0.03)	(0.008, 0.04)	(0.008, 0.04)
(16) rule on lang through Famcare	0.02 **	0.01	(0.006, 0.04)	(0.009, 0.04)	(0.009, 0.04)
**Panel C. Dependent variable is the child’s motor score**					
(17) safe on motor through Famcare	−0.005	0.006	(−0.01, 0.007)	(−0.02, 0.005)	(−0.02, 0.005)
(18) self_care on motor through Famcare	0.01 **	0.008	(0.002, 0.03)	(0.002, 0.03)	(0.003, 0.03)
(19) word on motor through Famcare	0.01	0.006	(−0.003, 0.02)	(−0.003, 0.02)	(−0.003, 0.03)
(20) read on motor through Famcare	0.02 ***	0.007	(0.008, 0.03)	(0.01, 0.04)	(0.01, 0.04)
(21) sing on motor through Famcare	0.01 **	0.008	(0.004, 0.03)	(0.004, 0.04)	(0.004, 0.04)
(22) play on motor through Famcare	0.01 **	0.008	(0.001, 0.03)	(0.001, 0.03)	(0.001, 0.03)
(23) getalong on motor through Famcare	0.02 **	0.007	(0.007, 0.03)	(0.007, 0.04)	(0.007, 0.04)
(24) rule on motor through Famcare	0.02 **	0.008	(0.007, 0.03)	(0.007, 0.04)	(0.007, 0.04)
**Panel D. Dependent variable is the child’s social-emotional score**					
(25) safe on soemo through Famcare	−0.007	0.009	(−0.02, 0.01)	(−0.03, 0.008)	(−0.03, 0.008)
(26) self_care on soemo through Famcare	0.02 **	0.009	(0.005, 0.04)	(0.003, 0.04)	(0.003, 0.04)
(27) word on soemo through Famcare	0.01	0.009	(−0.005, 0.03)	(−0.005, 0.04)	(−0.006, 0.04)
(28) read on soemo through Famcare	0.03 ***	0.01	(0.01, 0.05)	(0.01, 0.05)	(0.01, 0.05)
(29) sing on soemo through Famcare	0.02 **	0.01	(0.006, 0.04)	(0.004, 0.04)	(0.004, 0.04)
(30) play on soemo through Famcare	0.02 **	0.01	(0.001, 0.04)	(0.002, 0.05)	(0.002, 0.05)
(31) getalong on soemo through Famcare	0.02 ***	0.009	(0.01, 0.04)	(0.005, 0.04)	(0.005, 0.04)
(32) rule on soemo through Famcare	0.03 ***	0.01	(0.009, 0.05)	(0.01, 0.05)	(0.01, 0.04)

Notes: The estimates were based on the SEM with control variables. The independent variables were all observed parenting information measures (safe, self_care, word, read, sing, play, getalong, and rule). The definitions of these measures are shown in Table 1. The mediator was the latent variable of family care (Famcare). *** *p* < 0.01; ** *p* < 0.05.

**Table 5:** Estimates of indirect effects of parenting information on child development through different components of family care.

Indirect Effect	Point Estimate	Bootstrap S.E.	95% CI (Percentile)	95% CI (BC)	95% CI (BCa)
	(1)	(2)	(3)	(4)	(5)
**Panel A. Dependent variable is the child’s cognitive score.**					
(1) Parinfor on cog through soutoy	0.005	0.004	(−0.002, 0.01)	(−0.002, 0.02)	(−0.002, 0.02)
(2) Parinfor on cog through vartoy	0.02 ***	0.007	(0.01, 0.04)	(0.02, 0.04)	(0.02, 0.04)
(3) Parinfor on cog through playact	−0.008	0.007	(−0.02, 0.006)	(−0.02, 0.007)	(−0.02, 0.007)
(4) Parinfor on cog through book	0.004	0.005	(−0.006, 0.01)	(−0.003, 0.01)	(−0.003, 0.01)
(5) Parinfor on cog through magz	0.004	0.003	(−0.002, 0.01)	(−0.001, 0.009)	(−0.002, 0.009)
**Panel B. Dependent variable is the child’s language score.**					
(6) Parinfor on lang through soutoy	0.001	0.003	(−0.005, 0.007)	(−0.004, 0.006)	(−0.007, 0.006)
(7) Parinfor on lang through vartoy	0.02 ***	0.006	(0.01, 0.03)	(0.01, 0.03)	(0.01, 0.04)
(8) Parinfor on lang through playact	0.01	0.008	(−0.005, 0.03)	(−0.004, 0.02)	(−0.003, 0.02)
(9) Parinfor on lang through book	0.003	0.005	(−0.006, 0.01)	(−0.01, 0.01)	(−0.01, 0.01)
(10) Parinfor on lang through magz	0.004	0.004	(−0.003, 0.01)	(−0.003, 0.01)	(−0.003, 0.01)
**Panel C. Dependent variable is the child’s motor score.**					
(11) Parinfor on motor through soutoy	0.002	0.003	(−0.004, 0.009)	(−0.003, 0.01)	(−0.006, 0.01)
(12) Parinfor on motor through vartoy	0.02 ***	0.006	(0.01, 0.03)	(0.01, 0.03)	(0.01, 0.03)
(13) Parinfor on motor through playact	0.004	0.006	(−0.008, 0.02)	(−0.008, 0.01)	(−0.008, 0.01)
(14) Parinfor on motor through book	0.005	0.005	(−0.004, 0.01)	(−0.004, 0.01)	(−0.005, 0.01)
(15) Parinfor on motor through magz	0.001	0.003	(−0.004, 0.007)	(−0.003, 0.007)	(−0.003, 0.008)
**Panel D. Dependent variable is the child’s social-emotional score.**					
(16) Parinfor on soemo through soutoy	−0.001	0.003	(−0.007, 0.006)	(−0.007, 0.005)	(−0.008, 0.005)
(17) Parinfor on soemo through vartoy	0.02 ***	0.005	(0.007, 0.03)	(0.007, 0.03)	(0.007, 0.03)
(18) Parinfor on soemo through playact	0.03 ***	0.007	(0.02, 0.04)	(0.02, 0.04)	(0.02, 0.05)
(19) Parinfor on soemo through book	0.01 **	0.005	(0.002, 0.02)	(0.002, 0.02)	(0.001, 0.02)
(20) Parinfor on soemo through magz	−0.0002	0.003	(−0.006, 0.006)	(−0.007, 0.004)	(−0.01, 0.004)

Notes: The estimates were based on the SEM with control variables. The independent variable was the latent variable of parenting information (Parinfor). The mediators were all observed family care measures (soutoy, vartoy, playact, book, and magz). The definitions of these measures are shown in Table 1. *** *p* < 0.01; ** *p* < 0.05.

## Appendix A. Survey Instruments

**Table A1:** Parenting information questionnaire.

Item	Cronbach’s Alpha
1. The caregiver was told about how to teach the child to keep away from danger (such as pesticides, pond, and fire).	0.87
2. The caregiver was told about how to teach the child to self-care (such as brushing teeth and wearing clothes).	0.86
3. The caregiver was told about how to teach the child to understand and to use words.	0.86
4. The caregiver was told about how to read books with the child.	0.87
5. The caregiver was told about how to sing songs with the child.	0.86
6. The caregiver was told about how to play games with the child.	0.85
7. The caregiver was told about how to teach the child to get along with peers.	0.86
8. The caregiver was told about how to teach the child to understand and to obey the rules.	0.86
**Total**	**0.88**

**Table A2:** Family Care Indicators (FCIs).

Subscale	Cronbach’s Alpha
**Sources of play materials**	
1. Home-made toys.	0.74
2. Household objects.	0.75
3. Things from outside.	0.75
4. Toys bought from store.	0.74
**Varieties of play materials**	
5. Things which make/play music.	0.73
6. Things for drawing/writing.	0.74
7. Picture books (not school-books).	0.73
8. Things meant for stacking, con structing, building (blocks).	0.73
9. Things for moving around (balls, bats, etc.).	0.73
10. Toys for learning shapes and colors.	0.73
11. Things for pretending (dolls, tea-set, etc.).	0.74
**Play activities**	
12. Read books or look at picture-books with child.	0.74
13. Tell stories to child.	0.74
14. Sing songs with child.	0.73
15. Take child outside home place.	0.74
16. Play with the child with toys.	0.73
17. Spend time with child in naming things, counting, drawing.	0.74
**Household books**	
18. Number of books in the home, excluding picture books for children.	0.75
**Magazines and newspapers**	
19. Number of magazines and newspapers in the home.	0.74
**Total**	**0.75**
